# Hypohydration produced by high-intensity intermittent running increases biomarkers of renal injury in males

**DOI:** 10.1007/s00421-021-04804-3

**Published:** 2021-09-15

**Authors:** Loris A. Juett, Katharine L. Midwood, Mark P. Funnell, Lewis J. James, Stephen A. Mears

**Affiliations:** School of Sport, Exercise and Health Sciences, Loughborough University, Leicestershire, LE11 3TU UK

**Keywords:** Renal injury, Water intake, Dehydration, NGAL, KIM-1

## Abstract

**Purpose:**

Whilst there is evidence to suggest that hypohydration caused by physical work in the heat increases renal injury, whether this is the case during exercise in temperate conditions remains unknown. This study investigated the effect of manipulating hydration status during high-intensity intermittent running on biomarkers of renal injury.

**Methods:**

After familiarisation, 14 males (age: 33 ± 7 years; V̇O_2peak_: 57.1 ± 8.6 ml/kg/min; mean ± SD) completed 2 trials in a randomised cross-over design, each involving 6, 15 min blocks of shuttle running (modified Loughborough Intermittent Shuttle Test protocol) in temperate conditions (22.3 ± 1.0 °C; 47.9 ± 12.9% relative humidity). During exercise, subjects consumed either a volume of water equal to 90% of sweat losses (EU) or 75 mL water (HYP). Body mass, blood and urine samples were taken pre-exercise (baseline/pre), 30 min post-exercise (post) and 24 h post-baseline (24 h).

**Results:**

Post-exercise, body mass loss, serum osmolality and urine osmolality were greater in HYP than EU (*P* ≤ 0.024). Osmolality-corrected urinary kidney injury molecule-1 (uKIM-1) concentrations were increased post-exercise (*P* ≤ 0.048), with greater concentrations in HYP than EU (HYP: 2.76 [1.72–4.65] ng/mOsm; EU: 1.94 [1.1–2.54] ng/mOsm; *P* = 0.003; median [interquartile range]). Osmolality-corrected urinary neutrophil gelatinase-associated lipocalin (uNGAL) concentrations were increased post-exercise (*P* < 0.001), but there was no trial by time interaction effect (*P* = 0.073).

**Conclusion:**

These results suggest that hypohydration produced by high-intensity intermittent running increases renal injury, compared to when euhydration is maintained, and that the site of this increased renal injury is at the proximal tubules.

## Introduction

The incidence of acute kidney injury (AKI; diagnosed using changes in serum creatinine) following prolonged endurance events (i.e. marathons and ultramarathons) is variable (Lipman et al. 2014; Hoffman and Weiss 2016; Mansour et al. 2017; Poussel et al. 2020), but has been reported to be as high as 85% (Kao et al. 2015), with some severe cases resulting in temporary dialysis (Hodgson et al. 2017).Whilst these studies are likely to have overestimated AKI incidence, due to issues with measuring serum creatinine in close proximity to exercise (Hodgson et al. 2017), rises in serum creatinine following these events have been accompanied by rises in urinary neutrophil gelatinase-associated lipocalin (uNGAL) and urinary kidney injury molecule-1 (uKIM-1) (McCullough et al. 2011; Lippi et al. 2012; Mansour et al. 2017; Poussel et al. 2020), which are both novel biomarkers that indicate renal tubular injury (Kashani et al. 2017). The long-term consequences of exercise-associated increases in biomarkers of renal injury are not understood (Hodgson et al. 2017), but there is evidence for a link between AKI and chronic kidney disease (CKD) in a clinical setting (Coca et al. 2012).

The rise in biomarkers of renal injury reported following prolonged endurance events is likely due to several factors, including increases in sympathetic activity and body temperature, as well as muscle damage and hypohydration (Poortmans 1984; Junglee et al. 2013; Hoffman and Weiss 2016; Chapman et al. 2020). Of these factors, hypohydration may be of particular interest in the pathophysiology of exercise-associated renal injury, as whilst it is commonly seen during prolonged endurance events (Cheuvront and Haymes 2001), it can be mitigated with fluid consumption. Doing so may reduce the degree of renal injury, as hypohydration has the potential to contribute to renal injury via multiple mechanisms (Bragadottir et al. 2009), including a reduction in renal blood flow (Smith et al. 1952). It was recently shown that hypohydration produced by simulated physical work in the heat appeared to increase renal injury (compared to when euhydration was maintained with water ingestion) (Chapman et al. 2020). However, though there is observational evidence to suggest that hypohydration may contribute to AKI after a marathon (Mansour et al. 2019), causation cannot be inferred from such data, and thus whether hydration status influences renal injury during exercise in temperate conditions remains unknown and warrants investigation.

Hypohydration also commonly occurs during team sports (Nuccio et al. 2017), where athletes may be at a high risk of renal injury due to the high exercise intensity (Bongers et al. 2017), which can cause a large reduction in renal blood flow (Poortmans 1984) (likely via an increase in sympathetic activity and core body temperature), and may, therefore, lead to ischaemia and subsequent renal injury (Basile et al. 2012). Moreover, the frequent changes of direction and velocity seen in team sports can cause muscle damage (Souglis et al. 2015). Increases in serum creatinine have been measured following soccer matches (Gravina et al. 2011; Colombini et al. 2014), but these increases may be due to the increased release of serum creatinine from damaged skeletal muscle and/or a temporary reduction in renal function, rather than intrinsic renal injury (Hodgson et al. 2017). Therefore, investigation using urinary markers of renal injury is warranted.

The aim of the present study was to investigate the effect of manipulating hydration status during high-intensity intermittent running in temperate conditions on uNGAL and uKIM-1 concentrations. It was hypothesised that hypohydration of ~ 2% body mass loss induced by high-intensity intermittent running would result in greater concentrations of uNGAL and uKIM-1, compared to when exercise was completed in a euhydrated state.

## Methods

### Subjects

Fourteen active (team sport/racquet sport players and/or runners) males (age 33 ± 7 years; height: 1.79 ± 0.06 m; body mass: 75.3 ± 7.6 kg; BMI: 23.6 ± 2.6 kg/m^2^; V̇O_2_peak: 57.1 ± 8.6 mL/kg/min) participated in this study. All subjects were healthy, non-smokers, with no previous kidney issues or illnesses. Exclusion criteria included regular use of anti-inflammatory medications or any current medical complications that may have impacted kidney function and/or the ability to complete the protocol. Whilst not all participants in the present study were team sport athletes, all participants were familiarised with the exercise protocol, so that it was not a novel stimulus for experimental trials. Ethical approval was granted by the Loughborough University Ethical Approvals (Human Participants) Sub-Committee.

### Study design

Subjects completed a preliminary trial, a familiarisation trial and two experimental trials in a randomised, cross-over design. Trials involved completing a modified version of the Loughborough Intermittent Shuttle Test (LIST) (Nicholas et al. 2000) with (EU trial) and without (HYP trial) water ingestion, followed by a ~ 20.5 h recovery period. Familiarisation and experimental trials were separated by ≥ 7 days.

### Preliminary trial

During this visit, subjects provided verbal and written informed consent, completed a health screen questionnaire and then had height and nude body mass (AFW-120 K, Adam Equipment Co., UK) measured. To determine V̇O_2_peak, subjects then performed an incremental running test on a motorised treadmill (Mercury h/p/cosmos, Nussdorf, Germany), involving sub-maximal and maximal phases. For the sub-maximal phase, the treadmill was initially set at a 1% incline and 8 km/h, increasing by 1 km/h every 4 min until heart rate exceeded 160 beats/min (Polar M400, Polar, Kempele, Finland). This final speed was used for the maximal phase, where the gradient started at 1% and increased by 1%/min, until volitional exhaustion. In the final minute of each sub-maximal stage and for the final minute of the maximal phase, expired gases were collected into a Douglas bag and analysed for oxygen (O_2_) and carbon dioxide (CO_2_) concentrations (Servomex 1400 Gas Analyzer, Servomex, Crowborough, UK), volume (Harvard Dry Gas 194 Meter, Harvard Apparatus Ltd, Edenbridge, UK) and temperature (RS Pro Digital Thermometer, RS components, Corby, UK). V̇O_2_ and V̇CO_2_ values were corrected using ambient air collected simultaneously (Betts and Thompson 2012).

### Familiarisation trial

Upon arrival, subjects provided a urine sample and nude body mass was measured, before they completed a standard warm-up on an indoor 20 m track (two repeats of: 3 min jogging, followed by three 20 m sprints, both at a self-selected pace, separated and followed by 2 min rest). Subjects then began the main exercise protocol, which was an adapted version of the LIST (the performance sprints included in a regular LIST were replaced by a cruise at ~ 95% V̇O_2peak_ to reduce trial variability in exercise intensity). This comprised of six 15 min blocks of exercise (90 min total) separated by 2 min rest, with a 10 min half-time rest between blocks three and four. Each block was paced using audio cues (Nicholas et al. 2000) and consisted of ~ 11 repeated cycles of the following sequence: 3 × walk (1.5 m/s), 1 × cruise (~ 95% V̇O_2_peak), 3 × jog (~ 55% V̇O_2_peak) and 3 × cruise. Upon completing the adapted LIST, subjects rested in a seated position for 30 min before a blood sample was taken. Subjects then provided a urine sample and nude body mass was measured. Throughout the familiarisation trial, subjects consumed water ad libitum*,* which was weighed and added to body mass change (pre-exercise mass minus post-exercise mass) to allow prescription of fluid intake in EU (90% of familiarisation trial sweat losses).

### Pre-trial standardisation

The day prior to their first experimental trial, subjects were instructed to consume at least 40 mL/kg body mass of fluid (marked water bottles were supplied to aid compliance) and to complete a 24 h food/fluid intake diary. Subjects were asked to replicate this in the 24 h before their second experimental trial. They were also asked to refrain from alcohol intake and strenuous exercise the day before trials. Subjects were sent reminders regarding pre-trial standardisation two days before experimental trials and all subjects confirmed they had adhered to pre-trial requirements upon arrival for experimental trials.

### Experimental trials

Subjects reported to the laboratory between 6 and 10 am, after an overnight fast (≥ 10 h without food or fluid). To control diurnal effects, trial start time was standardised within subjects. Upon arrival, subjects sat for 30 min before a blood sample was taken, during which they completed subjective feelings questionnaires (0–10 numbered scale; 0 = no symptom; 10 = maximum symptom) for headache, nausea, dizziness, thirst, gastrointestinal (GI) comfort, GI bloating, stomach fullness and urge to vomit. Thermal sensation was measured on a scale of − 10 (extremely cold) to +10 (extremely hot). After the blood sample, subjects provided a urine sample and their nude body mass was measured, before they consumed 3 mL water/kg body mass over 15 min. In EU, an additional water bolus was also provided at this time (15% of sweat losses in the familiarisation trial).

Subjects completed the standardised warm-up, then began the modified LIST. In EU, subjects consumed water equivalent to 10% of their sweat losses from the familiarisation in the rest between the warm-up and the LIST and after each block of the LIST, with an extra 5% at half-time (15%). In HYP, to help reduce the unpleasant effects of mouth dryness, subjects consumed 25 mL water in the rest periods after the first, third and fifth blocks. Heart rate was continuously monitored and then averaged for each block. Rating of perceived exertion (RPE; 6–20), ambient temperature and relative humidity (Kestrel 4400, Nielsen-Kellerman Co, Boothwyn, USA) were measured immediately after each block and subjective feelings questionnaires were completed after block 3.

Upon completion of the LIST, subjects rested in a seated position for 30 min and repeated the subjective feelings questionnaires before a blood sample was taken, a urine sample was collected, and nude body mass was measured. Subjects then left the laboratory with food weighing scales and were asked to record their ad libitum food and fluid intake for the remainder of the day. This was subsequently analysed using online software (Nutritics 2019, Dublin, Ireland). The following morning, subjects returned to the laboratory in a fasted state (≥ 10 h fast), 24 h post-baseline subjective feelings questionnaires were completed, blood/urine samples were collected, and nude body mass was measured.

### Sample analysis

Blood samples were collected by venepuncture of an antecubital vein. From each sample, 1 mL was dispensed into a tube containing K_2_ EDTA (1.75 mg/L, Teklab, Durham, UK) and used to determine haemoglobin concentration (cyanmethaemoglobin method) and haematocrit (microcentrifugation; Hawksley Microhematocrit Centrifuge, Hawksley, Worthing, UK), which were used to calculate changes in plasma volume relative to baseline (Dill and Costill 1974). The remaining blood was dispensed into a 5 mL tube containing K_2_ EDTA (1.6 mg/L, Sarstedt Ltd, Leicester, UK), which was stored on ice, and a 4.5 mL tube containing a clotting catalyst (Sarstedt Ltd, Leicester, UK). These were allowed to stand for a minimum of 20 min before being separated by centrifugation (2200 g, 15 min, 4 °C). The resulting plasma and serum were stored at − 80 °C. All urine samples were measured for volume and osmolality (Osmocheck; Vitech Scientific, Horsham, UK), before being stored at − 80 °C.

Serum samples were thawed and analysed for osmolality, using freezing-point depression (Osmomat Auto, Cryoscopic Osmometer, Gonotec, Berlin, Germany). The concentrations of creatinine, albumin, creatine kinase (CK), lactate dehydrogenase (LDH) and myoglobin in serum samples were determined using a bench-top analyser (ABX Pentra C400; Horiba medical, Northampton, UK). ELISAs were performed for plasma/urinary NGAL (Human NGAL ELISA Kit, BioPorto, Hellerup, Denmark) and urinary KIM-1 (KIM-1 Human ELISA Kit, Enzo Life Sciences, Lausen Switzerland), according to manufacturer’s instructions. Intra-assay coefficients of variation for plasma NGAL, uNGAL and uKIM-1 were 8.7, 8.8 and 6.8%, respectively.

### Data and statistical analysis

To control for potential effects caused by shifts in body water, relevant plasma/serum markers were corrected for changes in plasma volume. Furthermore, to control for urine concentration, urinary biomarkers were corrected for urine osmolality. In both cases, uncorrected and corrected data were analysed statistically and presented. Due to an error in data collection, data on subjective feelings questionnaires are presented as *n* = 12. Ad-libitum food and fluid intake data are presented as *n* = 11, as three subjects did not complete their diet diaries in sufficient detail to be accurately analysed.

Data analyses were performed using SPSS (version 23, SPSS, Chicago, USA). Data were checked for normality of distribution using a Shapiro–Wilk test. Data containing one factor (ad libitum food and fluid intake) were analysed using a paired *t*-test or Wilcoxon signed-rank test, as appropriate. A two-way repeated measures ANOVA was performed to analyse data containing two factors (hydration status × time; trial order × time). The Greenhouse–Geisser estimate was used to correct the degrees of freedom where the assumption of sphericity was violated. Significant ANOVA effects were followed up by post hoc paired *t*-tests or Wilcoxon signed-rank tests, as appropriate. Family-wise error rate was controlled using the Holm–Bonferroni adjustment. Datasets were determined to be significantly different when *P* ≤ 0.05. Normally distributed data are presented as (mean ± SD), and non-normally distributed data are presented as [median, interquartile range]. At the time of designing the present study, to our knowledge, there were no published data to inform the effect size of manipulating hydration status on uNGAL or uKIM-1 concentrations. Therefore, a power calculation performed in a prior study, that investigated the effect of muscle damage on uNGAL concentrations, indicated that 12 subjects would be required to detect the smallest relevant change, with a statistical power of 0.8 and an alpha of 0.05 (Junglee et al. 2013). This power calculation is also in agreement with more recently published data (Chapman et al. 2020).

## Results

### Trial conditions

Pre-exercise, body mass (HYP: 74.74 ± 7.79 kg, EU 74.83 ± 7.54 kg; *P* = 0.661), serum osmolality (HYP: 292 ± 2 mOsm/kgH_2_O, EU: 292 ± 3 mOsm/kgH_2_O; *P* = 0.911), haemoglobin (HYP: 15.8 ± 0.8 g/dL, EU: 15.9 ± 0.9 g/dL; *P* = 0.559), haematocrit (HYP: 43.1 ± 1.8%, EU 43.3 ± 1.9%; *P* = 0.739) and thirst sensation (HYP: 4 [3–6], EU 5 [3–6]; *P* = 0.926) were not different between trials, suggesting a similar hydration status at the start of trials. Ambient temperature (HYP: 22.2 ± 1.0 °C, EU: 22.3 ± 0.8 °C; *P* = 0.867) and relative humidity (HYP: 46.1 ± 14.1%, EU: 49.8 ± 12.2%; *P* = 0.144) were not different between the trials.

### Hydration status measurements

**Fig. 1:**
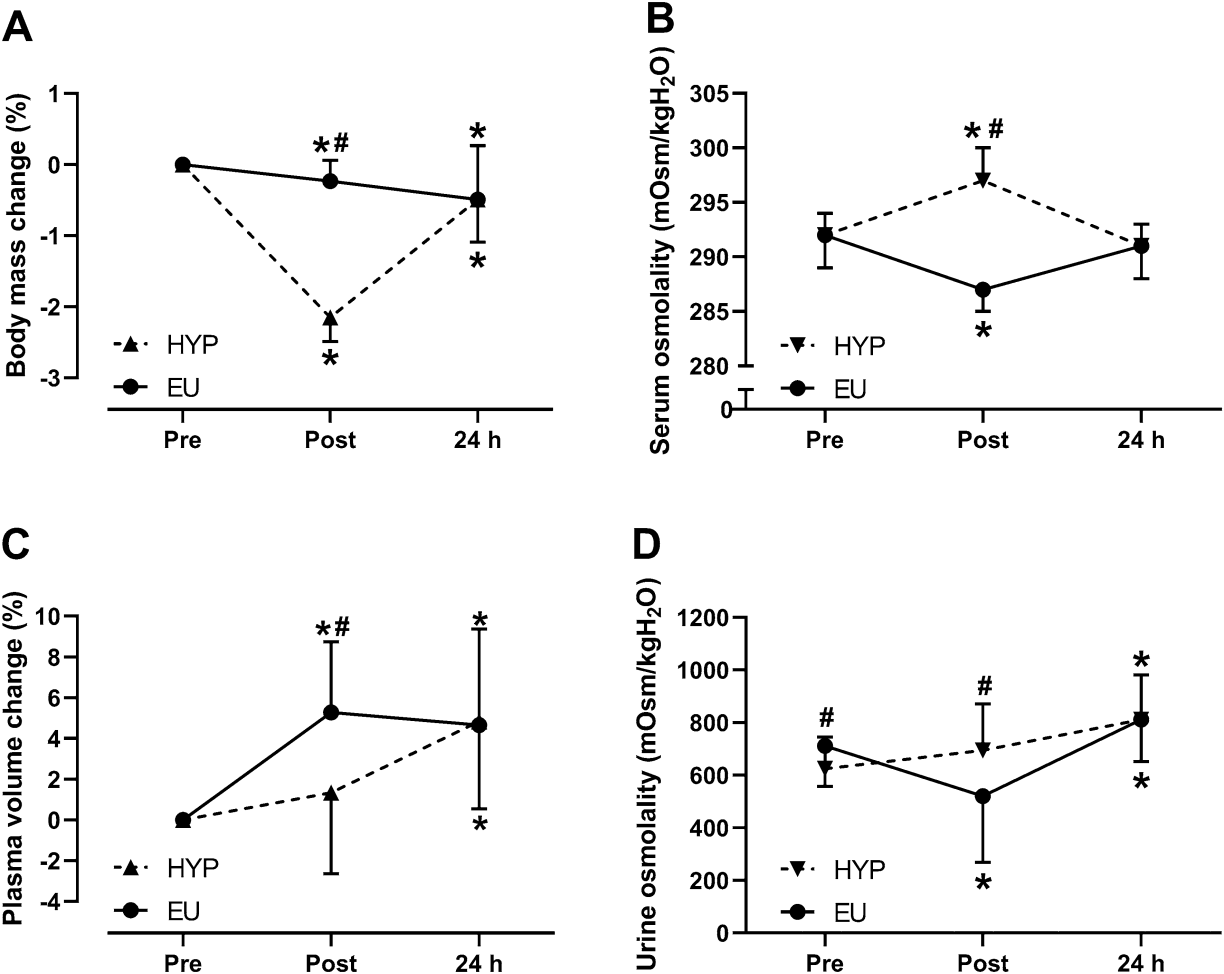
**A** change in body mass, **B** serum osmolality, **C** plasma volume change and **D** urine osmolality from pre-exercise (pre/baseline) to 30 min post-exercise (post) to 24 h post-baseline (24 h) in HYP (*n* = 14) and EU (*n* = 14). Statistical analyses from two-way repeated measures ANOVA, followed by Holm–Bonferroni-corrected paired *t*-tests. *Significantly different from pre, ^#^Significant difference between HYP and EU. Data are presented as mean ± SD

There was a trial by time interaction effect for changes in body mass (Fig. 1A; *P* < 0.001), serum osmolality (Fig. 1B; *P* < 0.001), plasma volume (Fig. 1C; *P* = 0.013) and urine osmolality (Fig. 1D; *P* = 0.001). Post-exercise, serum osmolality (*P* < 0.001) and urine osmolality (*P* = 0.024) were greater in HYP than EU, but plasma volume was greater in EU than HYP (*P* = 0.002). Sweat loss from pre- to post-exercise was not different between the trials (HYP: 1.82 ± 0.25 kg, EU: 1.82 ± 0.31 kg; *P* = 0.932). However, post-exercise, body mass loss was greater in HYP compared to EU (HYP: 1.6 ± 0.23 kg, EU: 0.17 ± 0.22 kg; *P* < 0.001), as 90% of sweat losses were replaced with water ingestion in EU (1639 ± 262 mL; excluding 3 mL/kg body mass bolus pre-exercise). In both trials, body mass (Fig. 1A) decreased from baseline to post-exercise (*P* ≤ 0.020) and remained decreased at 24 h post-baseline (*P* ≤ 0.033). Serum osmolality (Fig. 1B) increased from baseline to post-exercise in HYP (*P* < 0.001) and decreased in EU (*P* < 0.001) but returned to baseline levels at 24 h in both trials (*P* ≥ 0.063). Plasma volume (Fig. 1C) did not change from baseline to post-exercise in HYP (*P* = 0.229) but increased in EU (*P* < 0.001). At 24 h, plasma volume was elevated from baseline in both conditions (HYP: *P* = 0.002, EU: *P* = 0.003). Urine osmolality (Fig. 1D) decreased from baseline to post-exercise in EU (*P* = 0.003) but did not change in HYP (*P* = 0.131). At 24 h, urine osmolality was elevated from baseline in both trials (*P* ≤ 0.004).

### Biomarkers of renal injury

**Fig. 2:**
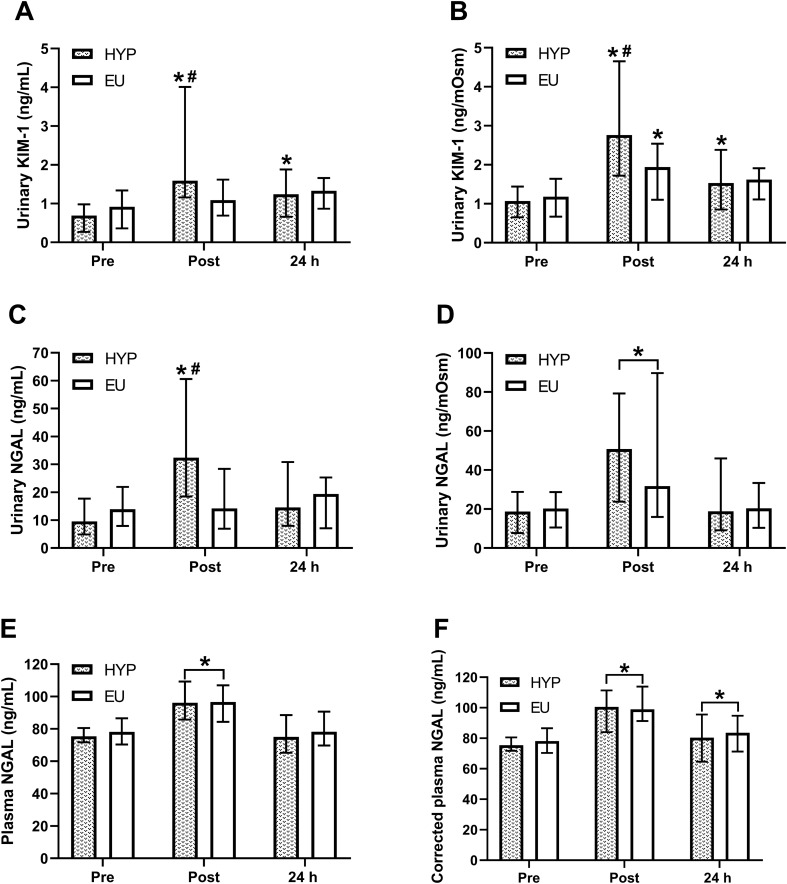
Urinary KIM-1 (**A**), osmolality-corrected urinary KIM-1 (**B**), urinary NGAL (**C**), osmolality-corrected urinary NGAL (**D**), raw plasma NGAL (**E**) concentrations and plasma NGAL concentrations corrected for plasma volume changes (**F**) before exercise (pre/baseline), 30 min post-exercise (post) and 24 h post-baseline (24 h) HYP (*n* = 14) and EU (*n* = 14). Statistical analyses from two-way repeated measures ANOVA, followed by Holm–Bonferroni-corrected paired *t*-tests or Wilcoxon signed-rank tests, as appropriate. *Significant difference from pre; ^#^significant difference between HYP and EU. Data are presented as median with interquartile range

There were trial by time interaction effects (*P* ≤ 0.014) for uKIM-1 (Fig. 2A) and uNGAL (Fig. 2C) concentrations, with both increased from baseline to post-exercise in HYP (uKIM-1: 2.3-fold increase, uNGAL: 3.4-fold increase; *P* = 0.002) but not in EU (*P* ≥ 0.300). Post-exercise, uKIM-1 and uNGAL were both greater in HYP compared to EU (uKIM-1: *P* = 0.012; uNGAL: *P* = 0.048). At 24 h, uKIM-1 concentrations were elevated from baseline in HYP (*P* = 0.002) but not EU (*P* = 0.230), whereas uNGAL concentrations at 24 h were not different from baseline in either trial (*P* ≥ 0.140). When correcting uKIM-1 for urine osmolality (Fig. 2B), there was a trial by time interaction effect (*P* = 0.011), with post-exercise osmolality-corrected uKIM-1 42% greater in HYP compared to EU (*P* = 0.003). In both trials, osmolality-corrected uKIM-1 increased from baseline to post-exercise (HYP: 2.6-fold increase, EU: 1.6-fold increase; *P* ≤ 0.048), remaining elevated at 24 h in HYP (*P* = 0.030) but returning to baseline values at 24 h in EU (*P* = 0.382). Correcting uNGAL for urine osmolality (Fig. 2D) removed the trial by time interaction effect (*P* = 0.073) but created a time effect (*P* = 0.015), with osmolality-corrected uNGAL increasing from baseline to post-exercise (*P* < 0.001) and returning to baseline values at 24 h (*P* = 0.452). There was no trial by time interaction effect (*P* = 0.171) for plasma NGAL concentrations (Fig. 2E), but there was a time effect (*P* < 0.001), which increased from baseline to post-exercise (*P* < 0.001), returning to baseline values at 24 h (*P* = 0.649). Correcting plasma NGAL for plasma volume changes (Fig. 2F) suggested that plasma NGAL remained elevated at 24 h (*P* = 0.026) but did not alter the significance of any other results.

**Fig. 3:**
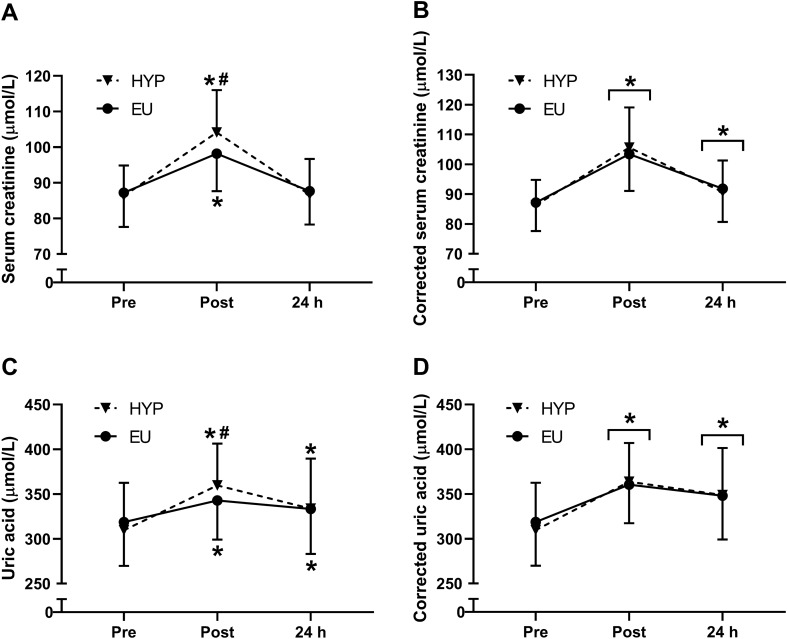
Raw serum creatinine (**A**) and serum creatinine corrected for plasma volume changes (**B**) before exercise (pre/baseline), 30 min post-exercise (post) and 24 h post-baseline in HYP (*n* = 14) and EU (*n* = 14). Statistical analyses from two-way repeated measures ANOVA, followed by Holm–Bonferroni-corrected paired *t*-tests. *Significant difference from pre; ^#^significant difference between HYP and EU. Data are presented as mean ± SD

There were trial by time interaction effects (*P* ≤ 0.008) for serum creatinine (Fig. 3A) and uric acid concentrations (Fig. 3C), which both increased from baseline to post-exercise in both trials (*P* < 0.001) and were greater in HYP than EU post-exercise (creatinine: *P* = 0.015; uric acid *P* = 0.042). At 24 h, creatinine concentrations returned to baseline in both trials (*P* ≥ 0.761), whereas uric acid concentrations remained elevated from baseline in both trials (*P* ≤ 0.030). Correcting creatinine (Fig. 3B) and uric acid (Fig. 3D) for plasma volume changes removed the trial by time interaction effect (*P* ≥ 0.234), but the time effect (*P* < 0.001) remained, with plasma volume corrected serum creatinine and uric acid increasing from baseline to post-exercise (*P* < 0.001) and remaining elevated at 24 h (*P* ≤ 0.005). When investigating effects of trial order, there were no trial effects (*P* ≥ 0.108) or trial by time interaction effects (*P* ≥ 0.084) for any of the biomarkers of renal injury measured, except plasma volume corrected uric acid (trial by time interaction effect: *P* = 0.047). However, with regards to plasma volume corrected uric acid, post-hoc tests revealed no significant differences between trials 1 and 2 at any time point (*P* ≥ 0.123), suggesting no effects of trial order on renal injury.

### Physiological response to exercise and perceptual measures

There was a trial effect for RPE (HYP: 15, 14–17, EU: 14, 13–15; *P* = 0.002) and heart rate (HYP: 149 ± 12 beats/min, EU: 143 ± 13 beats/min; *P* = 0.004). There was a trial by time interaction effect (*P* = 0.024) for serum albumin, which increased from baseline to post-exercise in HYP [765, 745–825 to 804, 781–903, *P* = 0.004] but not EU [779, 745–803 to 780, 766–799, *P* = 0.116], with 24 h concentrations not different to baseline in both trials [24 h HYP: 776, 737–818, 24 h EU: 763, 725–818, *P* ≥ 0.089). Post-exercise, serum albumin was greater in HYP than EU (*P* = 0.003).

There was a trial by time interaction effect (*P* < 0.001) for thirst (Table 1), with thirst greater in HYP than EU after block 3 of the LIST (*P* < 0.001) and post-exercise (*P* = 0.012). There was no trial by time interaction effect (*P* ≥ 0.123) for headache, nausea, dizziness or GI bloating (Table 1) and despite a trial by time interaction effect (*P* ≤ 0.037) for GI comfort, stomach fullness, urge to vomit and thermal sensation (Table 1), post hoc tests revealed no significant differences between trials at any time point (*P* ≥ 0.056).

**Table 1:** Subjective feelings questionnaires before exercise (pre/baseline), after the third block of the Loughborough Intermittent Shuttle Test (Block 3), post-exercise (post) and 24 h post-baseline in HYP (*n* = 12) and EU (*n* = 12)

	HYP				EU			
	Pre	Block 3	Post	24 h	Pre	Block 3	Post	24 h
Thirst (0–10)	4 [3–6]	6 [5–7]^#^	8 [6–9]^#^	3 [2–5]	5 [3–6]	2 [1–3]	3 [2–4]	2 [2–5]
Headache (0–10)	0 [0–0]	0 [0–0]	1 [0–2]	0 [0–0]	0 [0–0]	0 [0–0]	0 [0–0]	0 [0–1]
Nausea (0–10)	0 [0–0]	0 [0–1]	1 [0–2]	0 [0–0]	0 [0–0]	0 [0–0]	0 [0–1]	0 [0–0]
Dizziness (0–10)	0 [0–0]	1 [0–2]	1 [0–3]	0 [0–0]	0 [0–0]	0 [0–0]	0 [0–1]	0 [0–0]
GI bloating (0–10)	0 [0–0]	0 [0–0]	0 [0–1]	0 [0–0]	0 [0–0]	1 [0–1]	0 [0–2]	0 [0–0]
GI comfort (0–10)	0 [0–1]	1 [0–1]	1 [0–3]	0 [0–1]	1 [0–1]	0 [0–1]	0 [0–2]	0 [0–0]
Stomach fullness (0–10)	0 [0–1]	0 [0–1]	0 [0–0]	0 [0–1]	1 [0–1]	2 [0–2]	1 [0–2]	0 [0–2]
Urge to vomit (0–10)	0 [0–0]	0 [0–0]	0 [0–0]	0 [0–0]	0 [0–0]	0 [0–1]	0 [0–0]	0 [0–0]
Thermal sensation (− 10 to 10)	0 [0–0]	4 [4–4]	5 [4–5]	0 [0–0]	0 [0–1]	4 [4–4]	4 [4–5]	0 [0–0]

Statistical analyses from two-way repeated measures ANOVA, followed by Holm–Bonferroni-corrected Wilcoxon signed-rank tests. ^#^Represents a significant difference between trials. Data are presented as median [interquartile range]

### Muscle damage

There were no trial effects (*P* ≥ 0.169) or trial by time interaction effects (*P* ≥ 0.167), but there were time effects (*P* ≤ 0.039) for serum myoglobin, LDH and CK concentrations (Table 2). Serum myoglobin and CK concentrations increased from baseline to post-exercise (*P* < 0.001) and remained elevated from baseline at 24 h (*P* ≤ 0.003). Correction for plasma volume changes did not alter the significance of any serum myoglobin or CK results. Serum LDH concentration increased from baseline to post-exercise (*P* < 0.001) but returned to baseline values at 24 h (*P* = 0.158). However, correction for plasma volume changes suggested that LDH remained elevated from baseline at 24 h (*P* = 0.001). When investigating effects of trial order, there were no trial effects (*P* ≥ 0.168) for serum myoglobin, LDH or CK and there were no trial by time interaction effects (*P* ≥ 0.051) for LDH or CK. Despite a trial by time interaction effect (*P* = 0.033) for myoglobin, post hoc tests revealed no significant differences between trials 1 and 2 at any time point (*P* ≥ 0.165), suggesting no effects of trial order on muscle damage.

**Table 2:** Myoglobin, lactate dehydrogenase (LDH), LDH corrected for plasma volume changes (C LDH) and creatine kinase (CK) concentrations pre-exercise (pre/baseline), 30 min post-exercise (post) and 24 h post-baseline (24 h) in HYP (*n* = 14) and EU (*n* = 14) trials

Biomarker	Pre HYP	Pre EU	Post HYP	Post EU	24 h HYP	24 h EU
Myoglobin (ng/mL)	35 [25–46]	27 [22–45]	139* [93–465]	141* [85–239]	50* [29–77]	42* [30–53]
LDH (U/L)	179 [167–184]	169 [155–194]	215* [203–232]	198* [186–235]	182 [167–203]	178 [157–197]
C LDH (U/L)	179 [167–184]	169 [155–194]	222* [209–230]	214* [205–241]	190* [171–211]	186* [166–193]
CK (U/L)	292 [154–460]	215 [105–440]	398* [237–864]	342* [198–584]	392* [255–1040]	428* [222–699]

Statistical analyses from two-way repeated measures ANOVA, followed by Holm–Bonferroni-corrected Wilcoxon signed-rank tests. *Represents a significant difference from pre. Data presented as median [interquartile range]

### Food and fluid intake

Post-exercise energy (HYP: 12,126 ± 1716 v EU: 11,871 ± 2459 kJ), protein (HYP: 134 ± 27 v EU: 133 ± 40 g), fat (HYP: 127 ± 36 v EU: 114 ± 43 g), carbohydrate (HYP: 303 ± 69 v EU: 317 ± 88 g), and sodium (HYP: 2768 ± 766 v EU: 2878 ± 892 mg) intake were not different between trials (*P* ≥ 0.345). Post-exercise, the intake of water from drinks was greater in HYP (2997 ± 843 v 2348 ± 1166 g; *P* = 0.01), but the intake of water from foods did not differ between trials (867 ± 248 v 928 ± 210 g; *P* = 0.444). Total water intake for the day was greater in EU (4165 ± 902 v 5180 ± 1,336 g; *P* = 0.001). In HYP, post-exercise ad libitum water intake (including water from foods) was equal to 245 ± 58% of post-exercise body mass loss.

## Discussion

The aim of the present study was to investigate the effect of manipulating hydration status during high-intensity intermittent running on biomarkers of renal injury. The main findings were that osmolality-corrected uNGAL and uKIM-1 were both elevated post-exercise, regardless of hydration status, and that post-exercise osmolality-corrected uKIM-1 was further increased when subjects were hypohydrated. These findings partially confirmed our hypothesis and suggest high-intensity intermittent running causes renal tubular injury and that hypohydration exacerbates this, even in the absence of heat stress. Therefore, this study presents novel data demonstrating that hypohydration exacerbates renal injury after just 90 min of exercise in temperate conditions (a common place exercise scenario world-wide).

Post-exercise, uKIM-1 concentrations were greater in the HYP than EUH, despite correction for urine osmolality. This indicates greater production of uKIM-1 in HYP, rather than a simple urine concentration effect, and suggests increased renal tubular injury in HYP. It could be argued that there was a trend for greater post-exercise concentrations of osmolality-corrected uNGAL when hypohydrated, but this effect was not significant. Therefore, the differential responses of these biomarkers may provide insight into the location of renal injury that was exacerbated by hypohydration, as KIM-1 expression is increased in response to proximal tubular injury (Ichimura et al. 1998; Han et al. 2002; Kashani et al. 2017), whereas the main contributor to a rise in uNGAL is thought to be an increase in NGAL synthesis in the distal nephron (Paragas et al. 2011; Helanova et al. 2014; Bongers et al. 2017, 2018). However, even if post-exercise osmolality-corrected uNGAL concentrations were significantly greater in HYP, this would not necessarily be indicative of an increase in injury to the distal nephron, as a decrease in the proximal tubular reabsorption of NGAL can also contribute to a rise in uNGAL (Kashani et al. 2017; Schlader et al. 2019). Therefore, these findings suggest that hypohydration produced by high-intensity intermittent running likely exacerbates renal proximal tubular injury. This is in line with previous research by Chapman et al. (2020), which demonstrated that hypohydration produced by 2 h of simulated physical work in the heat (~ 39.7 °C) caused an increase (compared to when water was consumed to maintain euhydration) in osmolality-corrected urinary insulin-like growth factor-binding protein 7, indicating an increase in proximal tubular injury.

In the present study, whilst hypohydration appeared to exacerbate proximal tubular injury, the post-exercise elevations of osmolality-corrected uNGAL and uKIM-1, regardless of hydration status, suggest that the high-intensity intermittent running itself increased renal injury. This was likely due to a reduction in renal blood flow, which was not directly measured in the present study but was evidenced by post-exercise increases in serum uric acid, serum creatinine (although these can be influenced by muscle damage) (Knochel et al. 1974) and plasma NGAL (Schaub and Parikh 2016; Schlader et al. 2019), as well as the previously documented inverse correlation between renal blood flow and heart rate (Poortmans 1984). Given the high-intensity nature of the running in the present study, increases in sympathetic activity (Poortmans 1984; Zouhal et al. 2008) and core body temperature (Radigan and Robinson 1949; Smith et al. 1952; Sato et al. 2019) were likely contributors to a reduction in renal blood flow, which may lead to ischaemia and subsequent renal injury (Basile et al. 2012; Sato et al. 2019). In addition, post-exercise increases in serum myoglobin and uric acid, which were likely caused by muscle damage, may have also contributed to renal tubular injury via a reduction in renal blood flow, as well as other mechanisms (Blomberg et al. 2004; Sánchez-Lozada et al. 2008; Basile et al. 2012; Petejova and Martinek 2014; Roncal-Jimenez et al. 2018). Interestingly, the responses of muscle damage biomarkers in the present study’s population were similar to those of elite soccer players following a competitive match (Souglis et al. 2015).

Whilst the high-intensity intermittent running in the present study appeared to increase renal injury (likely via a reduction in renal blood flow), the lack of difference between trials with regards to post-exercise plasma NGAL suggests that the exacerbation of injury to the proximal tubules by hypohydration was not mediated by a further decrease in renal blood flow (Schaub and Parikh 2016; Schlader et al. 2019). The exacerbation of proximal tubular injury by hypohydration may have, therefore, been mediated by the rise in serum osmolality in HYP. This is indicative of intracellular fluid loss, where serum hyperosmolality draws fluid out of the intracellular fluid compartment via osmosis (Cheuvront and Kenefick 2014; James et al. 2019). This triggers the release of arginine vasopressin, which has been shown to increase renal oxygen consumption, possibly exacerbating renal injury by causing renal ischaemia and subsequent renal ATP depletion (Bragadottir et al. 2009; Basile et al. 2012; Cheuvront and Kenefick 2014). Evidence to support this potential mechanism comes from research in mice (Roncal-Jimenez et al. 2017) and humans, with Mansour et al. (2019) observing that post-exercise copeptin (a stable surrogate for arginine vasopressin) concentrations were greater in marathon runners with AKI than those without AKI.

Typically, an increase in serum osmolality is accompanied by a decrease in plasma volume (James et al. 2019). However, in HYP of the present study, post-exercise plasma volume was not reduced. This may have been due to a high osmotic pressure in the vascular space, as high-intensity exercise has been shown to generate high concentrations of lactate and vasopressin (Mears and Shirreffs 2013). These, combined with hypo-osmotic sweat losses, likely caused the serum hyperosmolality, which appeared to draw fluid from the intracellular compartment to the extracellular compartment (Cheuvront and Kenefick 2014; James et al. 2019). In EU, there was the expected increase in plasma volume as a result of the decreased serum osmolality. There was likely the same osmotic response to exercise, however, the fluid provided, particularly the last bolus, was sufficient to decrease serum osmolality and result in a temporary increase in plasma volume post-exercise.

Though maintaining euhydration during high-intensity intermittent exercise appeared to attenuate proximal tubular injury, it is important to acknowledge that the consumption of large amounts of water can increase the risk of exercise-associated hyponatremia (Rosner and Kirven 2007), evidenced in EU of the current study by a decrease in serum osmolality due the ingestion of plain water. However, no subject’s serum osmolality decreased to a concentration that would be deemed as dangerous (< 280 mOsm/kgH_2_O; Sahay and Sahay 2014), indicating that the hydration protocol used in EU was safe. This hydration protocol was also well tolerated, as evidenced by the lack of difference between trials in perceptual measures related to GI comfort. The hydration protocol also appeared to reduce the physiological strain of the high-intensity intermittent exercise, as measured by the lower heart rates and RPEs in EU, and may therefore have translated into differences in performance if this had been measured (Funnell et al. 2019; James et al. 2019). Whilst the hydration protocol in the present study appeared to attenuate proximal tubular injury, games players will typically drink fluid ad libitum, often consuming less than 50% of their sweat losses (Garth and Burke 2013; Funnell et al. 2017). Therefore, future research should investigate the effect of this rate of fluid ingestion during exercise on biomarkers of renal injury.

At 24 h post-baseline, there was evidence to suggest a decrease in body water in both trials, as seen by the lower body mass and increased urine osmolality compared to baseline. It is possible that the lower body mass may be at least partially explained by incomplete glycogen resynthesis, due to impairment of resynthesis by muscle damage (Zehnder et al. 2004) and insufficient carbohydrate intake (~ 4 g/kg body mass between post-exercise and 24 h in both trials) (Krustrup et al. 2011). It is not clear whether this is the case, though, as muscle glycogen was not measured in the present study, and studies that have measured muscle glycogen resynthesis following soccer-type exercise have produced contrasting findings (Zehnder et al. 2001; Krustrup et al. 2011).

Even if glycogen resynthesis was incomplete, this would not explain the elevated urine osmolality, which is indicative of a decrease in total body water. This apparent body water deficit at 24 h is unlikely to be due to an insufficient volume of water consumed in the recovery period, as it was also seen in EU. Moreover, in the 20.5 h recovery period of HYP, subjects rehydrated with a water volume equal to 245% of their body mass losses, which is well in excess of the 150% that has been shown to achieve rehydration in prior studies (Shirreffs et al. 1996; Evans et al. 2017). However, as the majority of the literature has focussed on short-term rehydration (≤ 6 h) (Evans et al. 2017), the optimal volume of fluid required to achieve rehydration over a longer time period (e.g. 20.5 h) remains unknown. Nonetheless, a more likely explanation for the apparent body water deficit at 24 h is inadequate sodium intake (Sawka et al. 2007). It is recommended that athletes rehydrate with fluid that has a sodium concentration of approximately 40–50 mmol/L (Maughan and Leiper 1995; Merson et al. 2008), but in HYP of the present study, when post-exercise sodium intake was expressed relative to total water intake, the concentration was approximately 30 mmol/L, with a similar amount consumed in EU. Therefore, inadequate sodium intake may have resulted in insufficient fluid retention (Maughan and Leiper 1995; Merson et al. 2008; Evans et al. 2017), and thus may explain the apparent body water deficit seen at 24 h in both trials.

Given that osmolality-corrected uKIM-1 remained elevated at 24 h in HYP and that there was evidence of a deficit in total body water at 24 h, renal injury was still present and may have been more likely or further increased if another bout of exercise was subsequently performed as is likely for team sports players. However, to our knowledge, the effect of bouts of exercise on consecutive days on uNGAL and uKIM-1 has only been investigated by one study, and whilst these biomarkers did not accumulate after three bouts of exercise performed on consecutive days (Bongers et al. 2017), this exercise was prolonged walking, which likely poses a lower risk of renal injury than more vigorous exercise. Therefore, more research is required to determine the effect of repeated bouts of vigorous exercise on novel biomarkers of renal injury, and the potential influence of hydration status.

In the present study, urine osmolality was used to correct the concentrations of uNGAL and uKIM-1, to account for changes in urine concentration. This is one of a variety of corrections that have been used in this field of research, including urinary creatinine (Junglee et al. 2012; Lippi et al. 2012; Bongers et al. 2017, 2018; Mansour et al. 2019; Chapman et al. 2020; Poussel et al. 2020) and urinary cystatin C (Bongers et al. 2017, 2018). The variety of correction factors used in the literature, along with variations in baseline concentrations (particularly with regards to uKIM-1), mean making comparisons between studies is challenging. Nonetheless, the post-exercise uNGAL concentrations in the present study are lower than those seen after prolonged endurance events (i.e. marathons and ultramarathons), which appear to be the type of exercise that produces the highest post-exercise uNGAL concentrations (Juett et al. 2021). This may be because the average exercise intensity during these events is only slightly lower than the present study, but exercise duration is longer and higher levels of muscle damage appear to be produced (McCullough et al. 2011; Mansour et al. 2019). In the context of exercise-associated renal injury, urine osmolality may be the most appropriate correction factor, as urinary creatinine and cystatin C may be increased during exercise because of muscle breakdown and decreased proximal tubular reabsorption, respectively (Conti et al. 2006; Junglee et al. 2012; Bongers et al. 2018). Some authors have also corrected urinary kidney injury biomarkers for urine flow rate (Junglee et al. 2013; Chapman et al. 2019, 2020). Whilst it is a limitation of the present study that urine flow rate was not measured, urine osmolality is regarded as an appropriate correction (Bongers et al. 2018; Schlader et al. 2019) and is more likely to become widely utilised, due to difficulties with precisely determining urine flow rate in many settings (Schlader et al. 2019). Another limitation of the present study is that certain mechanistic variables, such as core body temperature, renal blood flow and vasopressin/copeptin, were not measured. However, the significant difference in post-exercise serum osmolality between trials means a difference in vasopressin between trials at this timepoint is extremely likely, as changes in serum osmolality during exercise are strongly correlated with changes in serum arginine vasopressin (Wade 1984).

In conclusion, the results from the present study suggest that high-intensity intermittent exercise may increase renal injury. Maintaining euhydration with water intake during this exercise was a safe and well-tolerated intervention that appeared to attenuate proximal tubular injury and physiological strain. However, the long-term effects of increases in biomarkers of renal injury following exercise are not well understood and should be the focus of future investigations.

## Data Availability

The datasets generated during the current study are available from the corresponding author on reasonable request.

## Abbreviations

(u)KIM-1: (Urinary) Kidney injury molecule-1
(u)NGAL: (Urinary) Neutrophil gelatinase-associated lipocalin
AKI: Acute kidney injury
ANOVA: Analysis of variance
ATP: Adenosine triphosphate
CK: Creatine kinase
CKD: Chronic kidney disease
GI: Gastrointestinal
LDH: Lactate dehydrogenase
LIST: Loughborough Intermittent Shuttle Test
Osm: Osmolality
RPE: Rating of perceived exertion
SD: Standard deviation
